# Determining accurate conformational ensembles of intrinsically disordered proteins at atomic resolution

**DOI:** 10.1063/4.0000825

**Published:** 2025-10-27

**Authors:** Kaushik Borthakur, Thomas R Sisk, Francesco P Panei, Massimiliano Bonomi, Paul J Robustelli

**Affiliations:** 1 Dartmouth College, Hanover, NH, USA; 2 Institut Pasteur, Paris, Paris, France

## Abstract

Determining accurate atomic resolution conformational ensembles of intrinsically disordered proteins (IDPs) is extremely challenging. Molecular dynamics (MD) simulations provide atomistic conformational ensembles of IDPs, but their accuracy is highly dependent on the quality of physical models, or force fields, used. In this work, we demonstrate how to determine accurate atomic resolution conformational ensembles of IDPs by integrating all-atom MD simulations with experimental data from nuclear magnetic resonance (NMR) spectroscopy and small-angle x-ray scattering (SAXS) with a simple, robust and fully automated maximum entropy reweighting procedure. We demonstrate that when this approach is applied with sufficient experimental data, IDP ensembles derived from different MD force fields converge to highly similar conformational distributions. The maximum entropy reweighting procedure presented here facilitates the integration of MD simulations with extensive experimental datasets and enables the calculation of accurate, force-field independent conformational ensembles of IDPs at atomic resolution.

